# Reduction in chromosome mobility accompanies nuclear organization during early embryogenesis in *Caenorhabditis elegans*

**DOI:** 10.1038/s41598-017-03483-5

**Published:** 2017-06-16

**Authors:** Ritsuko Arai, Takeshi Sugawara, Yuko Sato, Yohei Minakuchi, Atsushi Toyoda, Kentaro Nabeshima, Hiroshi Kimura, Akatsuki Kimura

**Affiliations:** 1 Cell Architecture Laboratory, Structural Biology Center, National Institute of Genetics, Mishima, 411-8540 Japan; 2 Data Centric Science Research Commons of the Research Organization of Information and Systems, Transdisciplinary Research Integration Center, Research Organization of Information and Systems, Tokyo, 105-0001 Japan; 3 Cell Biology Center, Institute of Innovative Research, Tokyo Institute of Technology, Yokohama, 226-8503 Japan; 4 Comparative Genomics Laboratory, National Institute of Genetics, Mishima, 411-8540 Japan; 5 Department of Cell and Developmental Biology, University of Michigan Medical School, Michigan, 48109-2200 USA; 6 Department of Genetics, School of Life Science, The Graduate University for Advanced Studies (Sokendai), Mishima, 411-8540 Japan; 7 Department of Anatomy and Histology, Fukushima Medical University, School of Medicine, Hikarigaoka, Fukushima 960-1295 Japan; 8 Research Center for the Mathematics on Chromatin Live Dynamics (RcMcD), Hiroshima University, Higashi, Hiroshima 739-8530 Japan

## Abstract

In differentiated cells, chromosomes are packed inside the cell nucleus in an organised fashion. In contrast, little is known about how chromosomes are packed in undifferentiated cells and how nuclear organization changes during development. To assess changes in nuclear organization during the earliest stages of development, we quantified the mobility of a pair of homologous chromosomal loci in the interphase nuclei of *Caenorhabditis elegans* embryos. The distribution of distances between homologous loci was consistent with a random distribution up to the 8-cell stage but not at later stages. The mobility of the loci was significantly reduced from the 2-cell to the 48-cell stage. Nuclear foci corresponding to epigenetic marks as well as heterochromatin and the nucleolus also appeared around the 8-cell stage. We propose that the earliest global transformation in nuclear organization occurs at the 8-cell stage during *C*. *elegans* embryogenesis.

## Introduction

Chromosomes are long polymers that store genetic information, consisting of DNA and various proteins. In eukaryotes, chromosomes are packed inside the cell nucleus in an organised manner during interphase. For example, chromosomes are packed in a hierarchical manner known as a fractal globule structure, in which neighbouring chromatin assembles to form units of higher order structures^1^. Chromosomal territories represent another level of chromatin organization, in which different chromosomes do not mix with each other inside the nucleus but rather tend to maintain specific locations or positions (e.g. the nuclear centre or periphery)^2^. This specific organization is established and maintained in differentiated cells, where it is thought to be important for characteristic gene expression profiles^3, 4^. In contrast, not much is known regarding chromosomal organization in undifferentiated cells. For example, is chromatin organization “reset” in germ cells? When and how do chromosomes organise during development? According to studies in embryonic stem (ES) cells^5, 6^, there may be no substantial differences in global chromatin organization between differentiated and undifferentiated cells.

*Caenorhabditis elegans* is an appropriate model organism for studying changes in nuclear organization during early embryogenesis. *C*. *elegans* embryos are transparent, and the entirety of embryogenesis can be observed under a microscope^7^. To characterise the state of chromosomal organization during *C*. *elegans* early embryogenesis, we designed an experiment to track the mobility of a pair of homologous chromosomal loci in live cells during interphase. For this purpose, we used a *lacO*–LacI system in which the bacterial operator sequence *lacO* is artificially inserted into a chromosome and the position of this sequence is visualised with a bacterial LacI protein fused to green fluorescent protein (GFP)^8^. This system has been previously used to reveal various features of chromosomal organization. During *C*. *elegans* development, tissue-specific promoters take non-random radial positions inside the nucleus upon activation^9^. The dynamics of homolog pairing during meiosis have also been characterised using this system in *C*. *elegans* ^10^ as well as in the fission yeast *Schizosaccharomyces pombe* ^11^. The mobilities of chromosomal loci increase upon DNA damage, likely contributing to the efficiency of homology searches^12^. The quantified mobilities of the chromosomes are often interpreted as a free diffusion within a sub-region of the nucleus^8, 13, 14^. Another interpretation is that the loci do not diffuse freely but show sub-diffusive movement due to the polymeric nature of chromosomes, as demonstrated in *Escherichia coli*, *Caulobacter crescentus*, and in *Saccharomyces cerevisiae* ^15, 16^.

To detect the earliest change in global chromatin organization inside the interphase nucleus, we tracked *lacO* loci inserted into the *C*. *elegans* genome from the 2- to the 48-cell stage. A quantitative analysis of the mean square change in distance (MSCD) revealed a significant reduction in chromosome mobility during this time. Live-cell imaging of epigenetic marks and heterochromatin provided cytological evidence that a global transformation in nuclear organization occurs around the 8-cell stage in *C*. *elegans* embryos.

## Results

### Live-cell tracking of *lacO* loci inserted into *C*. *elegans* chromosomes

**Figure 1:**
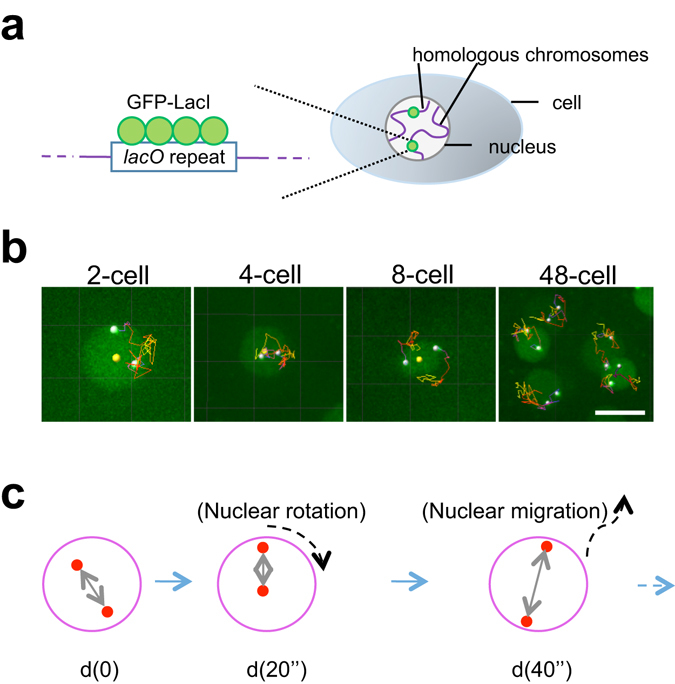
4D tracking analysis of *lacO* spots during *C*. *elegans* embryogenesis. (**a**) Schematic of the visualization of a pair of homologous loci in the *C*. *elegans* embryos. A *lacO* repeat was integrated into the *C*. *elegans* genome and detected by expression of the LacI protein fused to GFP. (**b**) Representative examples of *lacO* tracking at indicated stages. Two white dots in each panel show the *lacO* spots, and the yellow dot reveals the centre of the nucleus (not shown for the 48-cell stage). Lines show the trajectories of the *lacO* spots. Bar, 5 μm. (**c**) The distance between the two spots (*d*) reflects the mobility of the spots, excluding the effect of nuclear rotation and migration. *d*(0), *d*(20″), and *d*(40″) represent the distances between the two loci at time 0, 20, and 40 s, respectively.

We used the *lacO*–LacI system to visualise chromosomal loci in live cells (Fig. 1a). In addition to using the previously established strain AV221^10^, we established strain CAL0872, which contains a chromosomal *lacO* repeat and expresses the GFP::LacI protein under the control of the *pie-1* gene promoter. In AV221, the *lacO* repeat is present near the middle of chromosome *III*, which lacks the left end and is fused with chromosome *IV* ^10^. We identified the location of the *lacO* insertion in CAL0872 near the left end of chromosome *III* (Supplementary Fig. S1). In this study, we used these two strains, which harbour *lacO* repeats at different chromosomal locations, and focused on the features common to both strains.

Time-lapse imaging of loci was conducted using a spinning-disk confocal microscope with a different focal plane (1- or 2-μm intervals depending on the stage) every 20 s (Fig. 1b, Supplementary Movies S1–S4), and 3D tracking was performed using Imaris software. Phototoxicity due to imaging was not significant, as embryo hatching was confirmed after imaging, and loci mobilities did not change between the first and second halves of the imaging (Supplementary Fig. S2).

### Distribution of distances between loci consistent with random distribution before 8-cell stage but not later

We observed two GFP spots per nucleus because there is a pair of homologous chromosomes containing *lacO* in each nucleus. In this study, we focused on the distance between the two spots (Fig. 1c), as distance is not affected by either translational or rotational movements of the nucleus during imaging^14^.

**Figure 2:**
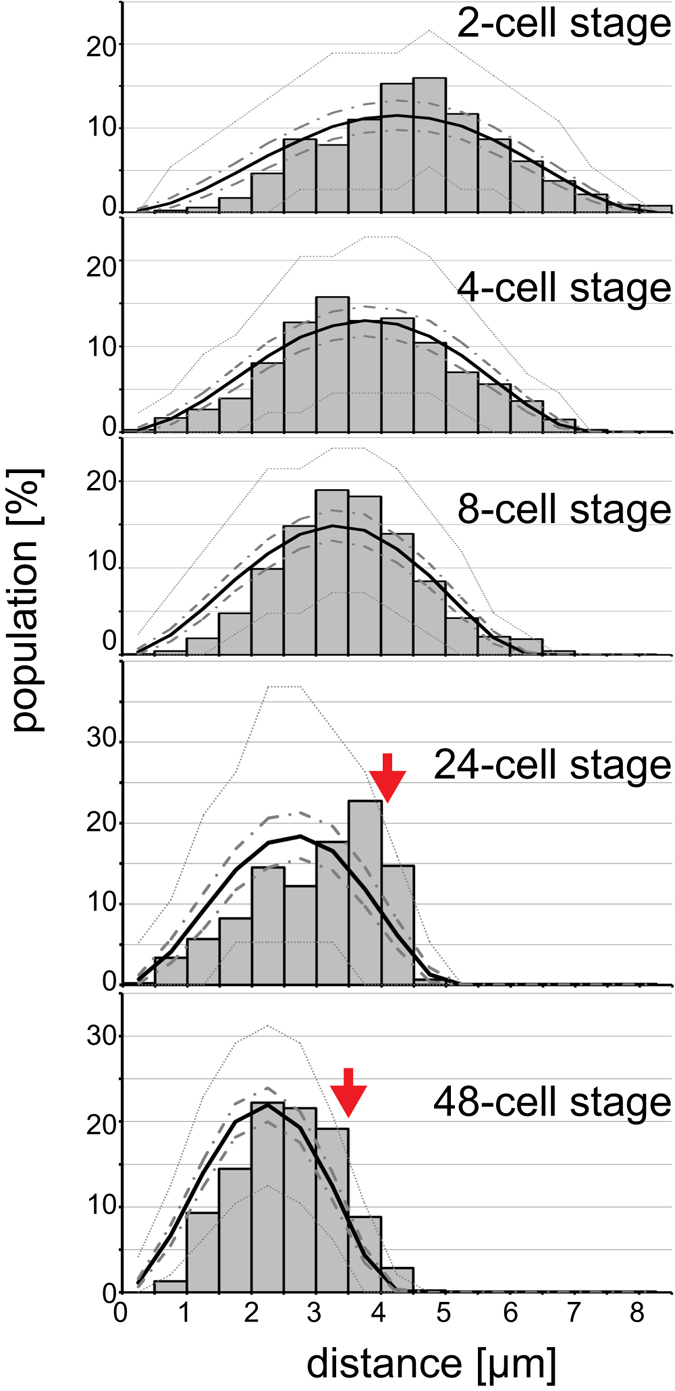
Distribution of distances between *lacO* loci. Histograms of the distances between the two *lacO* spots in each developmental stage. Sample sizes are indicated in Supplementary Table S3 (“the number of pairs of *lacO* spots”). Black solid lines indicate the expected distribution of the distances if the two spots were randomly positioned in a nucleus of the same size, predicted from a Monte Carlo simulation. The average sizes of the nuclei at each stage are described under “radius” in Supplementary Table S3. Thick dotted lines indicate the upper and lower limits of the 95% confidence interval calculated from a random simulation with the number of pairs equal to that of the *lacO* spots for each stage as described under “the number of pairs of the *lacO* spots” in Supplementary Table S3 (see Methods). Similarly, thin dotted lines indicate the limits when the number of random pairs was the same as the number of nuclei examined as described under “the number of nuclei” in Supplementary Table S3. The latter corresponds to an extreme situation where the two spots do not move at all in each nucleus, and thus the expected variation in the distribution is large. Red arrows indicate where the experimentally obtained distribution did not agree with the random position scenario. Differences in the distributions among different stages are more directly compared in Fig. S3, where the distances between the *lacO* spots were normalised by the radius of the nucleus.

We examined whether the distribution of distances was consistent with random positioning of chromosomes in the nucleus. First, we calculated the theoretical distribution of the distance between two spots randomly positioned in a sphere with a radius of the nucleus (Fig. 2, black line). The expected distribution was a bell shape with the mean equal to the radius. In cells at the 8-cell stage or earlier, the *in vivo* distribution was similar to the random distribution (Fig. 2). This result indicates that before the 8-cell stage, chromosomal positions are completely random.

In contrast, after the 8-cell stage, the random distribution did not account for the experimental distribution. Normalised distances between the two spots were biased toward longer distances in 24- and 48-cell embryos (Fig. 2, red arrows; Supplementary Fig. S3). This suggests that the chromosomes tend to be positioned near the nuclear periphery during these stages. This is consistent with a previous report that some chromosomal loci move to the nuclear periphery after development in *C*. *elegans* ^9^. Our current results suggest that this non-random nuclear organization is initiated after the 8-cell stage.

### Loci mobility decreases drastically from the 2-cell to the 48-cell stage

**Figure 3:**
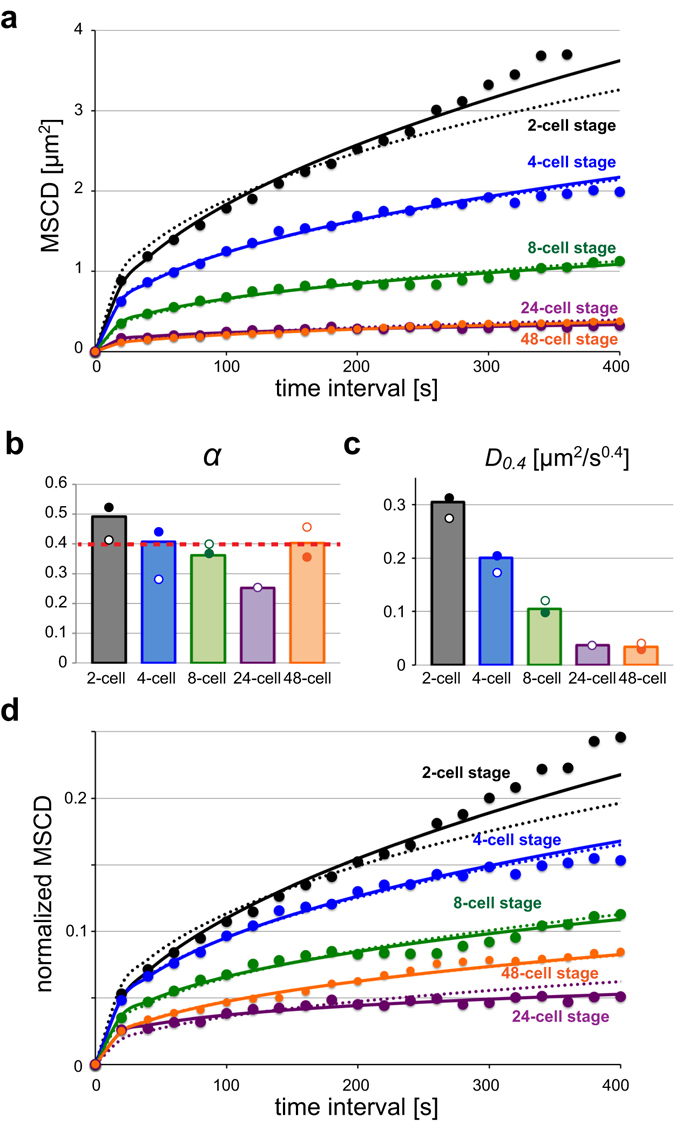
MSCD analyses of mobility. (**a**) MSCD was plotted against the time interval (τ) for each stage. Different colours indicate different stages. Solid lines indicate the best-fit curve in the form of MSCD = *D*_*α*_ × *τ* ^*α*^, and dotted lines indicate the best-fit curve in the form of MSCD = *D*_*0.4*_ × *τ* ^0.4^ (i.e. *α* was fixed at 0.40). (**b**) The optimum value of *α* when the data were fitted to MSCD = *D*_*α*_ × *τ* ^*α*^. *α* = 0.40 (red dotted line) was the optimum value when fitting all data, including different stages for both strains, with a common value of *α*. (**c**) The optimum value of *D*_*0.4*_ when the data was fitted to MSCD = *D*_*0.4*_ × *τ* ^0.4^. (**d**) The value of MSCD was normalised using the size of the nucleus by dividing MSCD by the average radius squared of the nucleus at each stage. For (**b**,**c**), bar graphs show the results using all data from the two strains, and filled and open circles indicate results from strains AV221 and CAL0872, respectively. We did not collect data for strain AV221 at the 24-cell stage.

We quantified the mobility of the pair of loci by calculating the MSCD^14^. MSCD is similar to the mean squared displacement (MSD), which is often used to quantify the mobility of an object inside the cell. We used MSCD rather than MSD because the MSCD can exclude the effect of the movement of the container (in this case, the cell nucleus) (Fig. 1c). When we calculated MSCD values and plotted them against the observation time intervals, *τ*, we observed a drastic reduction in mobility from the 2-cell stage to the 48-cell stage (Fig. 3a and Supplementary Fig. S4). In the 2-cell stage, the MSCD was larger than in other stages during the same time interval. This means that the loci were moving faster in the 2-cell stage than in the other stages, as the distance between the loci changed more.

The relationship between MSCD and *τ* is often formulated as MSCD = *D*_*α*_ × *τ* ^*α*^, where *α* and *D*_*α*_ are constant parameters. *α* is known to take the value 1 when the loci move according to Brownian motion (i.e. normal diffusion). *α* is less than 1 when the movements of the loci are constrained (“subdiffusion”). For example, in the case of a locus on a polymer, the locus cannot move freely because movement is restricted by the adjacent polymer regions. We fitted the experimental MSCD data obtained from each developmental stage to the formula MSCD = *D*_*α*_ × *τ* ^*α*^ (Fig. 3a and Supplementary Fig. S5, solid lines). The estimated value of *α* was about 0.4 in each stage (Fig. 3b). The mean *α* value (±S.D.) across all stages was 0.38 ± 0.09. When we calculated the mean *α* values separately for AV221 and CAL0872, they were 0.42 ± 0.08 and 0.36 ± 0.09, respectively. There was no significant difference between the two strains (Mann-Whitney *U*-test). Next, we conducted another fitting, but instead of assuming different values of *α* for different stages of embryogenesis, we assumed a common value of *α* for all stages. The fitted value of *α* common to all stages was 0.40. We confirmed that using *α* = 0.40, the formula fitted the experimental data well (Fig. 3a and Supplementary Fig. S5, dotted lines). This result indicates that, although the magnitude of the mobility differs across stages, the coefficient of *α* (~0.4) is a stage-independent feature of the chromosomes.

Because the common value of *α* = 0.40 can explain the MSCD at different stages, the stage-dependent change in MSCD should be reflected in the value of *D*_*0.4*_ in the formula MSCD = *D*_*0.4*_ × *τ* ^*0*.*40*^. The optimum value of *D*_*0.4*_ [μm^2^/s^0.4^] calculated from the fitting (Fig. 3a and Supplementary Fig. S5, dotted lines) decreased monotonically as embryogenesis proceeded (Fig. 3c), as expected.

One possible cause of the observed reduction in chromosomal mobility in later cell stages is the decrease in nuclear size. During embryogenesis, the size of the interphase nucleus becomes smaller over time^17^, which may saturate MSCD levels. To investigate this possibility, we normalised the MSCD relative to the nuclear size by dividing the MSCD by the squared length of the nuclear radius. The normalised MSCD also decreased as embryogenesis proceeded (Fig. 3d), indicating that nuclear size alone cannot account for the decrease in chromosomal mobility in the later stages of embryogenesis.

### Formation of foci representing epigenetic marks, heterochromatin, and the nucleolus after the 8-cell stage

**Figure 4:**
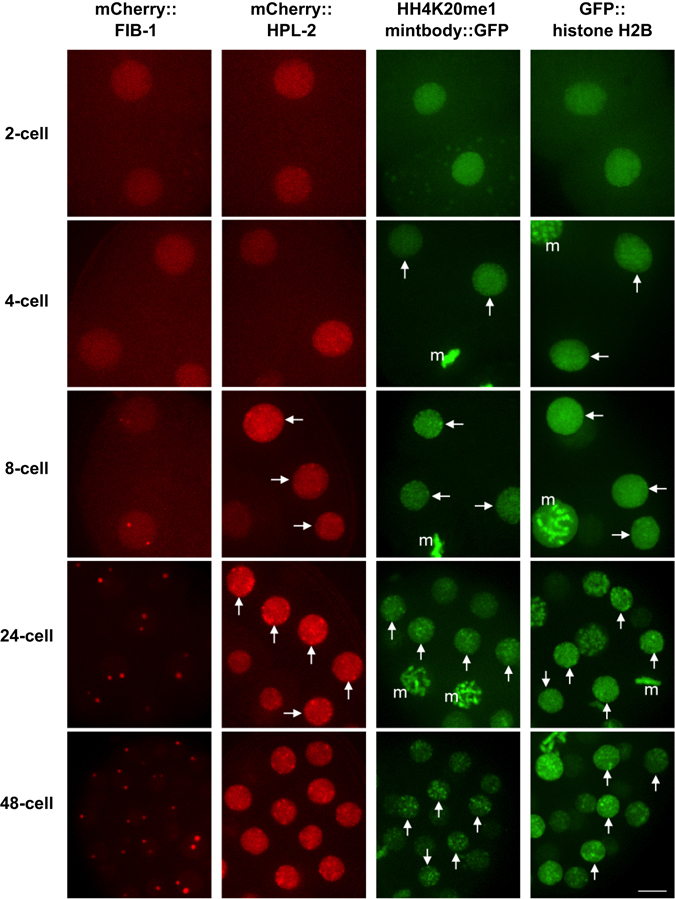
Live imaging of chromosomal domain formation during early embryogenesis. Imaging of FIB-1 (fibrillarin protein, nucleolus marker); HPL-2 (HP1 protein, heterochromatin marker); H4K20me1 mintbody (antibody fragment against mono-methylated lysine 20 of histone H4); and histone H2B. “m” indicates mitotic chromosomes or nucleus. Other signals are from interphase nuclei (arrows indicate representative nuclei). Bar, 5 μm.

The above quantitative analyses of the *lacO* loci indicated that global changes in chromosomal mobility occur during the 2- to 48-cell stages, most dramatically around the 8-cell stage. To investigate whether this physical change is linked to the molecular composition of the chromosomes, we cytologically observed the nucleus during these stages. Observation of histone H2B fused to GFP showed a slight increase in nuclear regions with dense signals (Fig. 4). To observe specific regions on the chromosomes, we constructed strains to visualise heterochromatin regions (mCherry-HPL2)^18^ and the nucleolus (mCherry-fibrillarin)^19^. For both heterochromatin and the nucleolus, signal was uniform throughout the nucleus in 2- and 4-cell stage embryos (Fig. 4). Foci became evident at the 8-cell stage and were present thereafter. For the nucleolus marker, almost all the signal became concentrated on several foci in the nucleus after the 24-cell stage, as reported previously^19, 20^. Both markers thus supported our observation of the global changes in chromosomal biochemical properties during these stages.

To further assess these global biochemical properties, we aimed to visualise epigenetic marks on histone proteins directly. Histone proteins are the major chromosomal protein components and can be post-translationally modified, which can be an indicator of different chromatin states^21^. Monoclonal antibodies against specific histone modifications are critical tools in epigenetic studies^22^. Recently, fragments of these specific antibodies have been fused to GFP (“mintbodies”) to visualise the probes inside the cell^23^. In this study, we utilised a mintbody against mono-methylation of Lys20 of histone H4 (H4K20me1)^24^. This modification is important for various aspects of chromatin function^25^, such as transcriptional repression^26^, chromatin compaction^27^, X-chromosome inactivation^28, 29^, and kinetochore assembly^30^. We have previously reported that this mintbody selectively localises to the X chromosomes in late embryonic stages^24^. In this study, we examined the localization of the mintbody during early embryogenesis. The signal of H4K20me1 was uniformly distributed during the 2- and 4-cell stages, whereas fluorescent foci started to form at the 8-cell stage (Fig. 4) and became clear in 24-cell-stage or later embryos. Visualization of epigenetic marks, heterochromatin, and the nucleolus thus all supported a global reorganization of chromosomes during early embryogenesis around the 8-cell stage in *C*. *elegans*.

## Discussion

In this study, we provide evidence that global changes in both the physical and biochemical properties of chromosomes occur around the 8-cell stage during *C*. *elegans* embryogenesis. At the earliest stages of development (e.g. 2-cell stage), there was no indication of any specific nuclear organization. Chromosomal positions at this stage could be explained by random positioning, chromosomal mobility was high, and no evident epigenetic marks or nuclear domains were observed. In contrast, after the 8-cell stage, chromosomal positions became non-random, chromosomal mobility decreased, and epigenetic marks and nuclear domains became evident. This nuclear reorganization may be related to gene expression. In *C*. *elegans*, zygotic transcription starts at the 4-cell stage^31^, and massive transcription becomes evident around the 8-cell stage^32^. This timing is consistent with our observation of nuclear reorganization. While we showed that the change in chromosomal mobility coincided with the formation of epigenetic marks and a nuclear domain, the causal relationship between these physical and biochemical changes is unclear. This represents an interesting question to be addressed in the future.

We did not observe significant changes in chromosomal mobility among different cell types at the same cell stages (Supplementary Fig. S4). For example, germ cells and somatic cells seemed to behave similarly in terms of chromosomal mobility. In *C*. *elegans*, transcription in germ cells is globally silenced^31^. This global silencing is caused by inhibition of transcriptional elongation through the regulation of RNA polymerase II activity. Therefore, differential gene expression among different cell types may not be regulated at the level of global chromosomal structure. However, specific loci may behave differently, even though the two randomly integrated arrays represent general chromosomal dynamics. It would be interesting to investigate whether highly transcribed loci in somatic cells show different mobilities in germ cells, which could be done by targeting *lacO* arrays to predefined loci.

Our quantification of MSCD revealed that the anomalous exponent of diffusion, *α*, was ~0.4 regardless of the developmental stage. To our knowledge, this is the first demonstration that the value of *α* is maintained at around 0.4 at different developmental stages in a multicellular organism. Considering that the value of *α* of the MSD is 0.4–0.5 in both *E*. *coli* and yeast (for both transcriptionally active and inactive states)^33^, an *α* of 0.4–0.5 may be a universal feature of chromosomes regardless of species. The physical basis of this universality is the polymeric nature of chromosomes^34^, and is explained by simulations based on a fractal globule model^35^. Another interpretation for the lack of an increase in MSCD against time interval is that, even though loci can move freely in a method similar to Brownian motion (*α* = ~1), movement at longer time intervals is restricted by steric hindrance owing to the size of the nucleus or the size of chromosomal territories. This idea is popular among studies involving the quantification of chromosomal mobility^14^. However, the present results do not indicate that this is the case. First, the value for the exponent *α* is ~0.4 and thus is obviously smaller than 1 even in the shortest time interval region (Fig. 3a and Supplementary Fig. S5). Second, the saturated MSCD is very small compared with the size of the nucleus. A simple simulation of random motion inside a sphere indicated that the normalised MSCD should reach ~0.26 if the nuclear boundary serves as an upper limit. However, the obtained MSCD values were much smaller than the upper limit value. Therefore, the lack of an increase in MSCD cannot be explained by the upper mobility limit determined by the nuclear region but instead results from the sub-diffusive nature of chromosomal mobility. MSCD and MSD values measured in other systems should be assessed for whether they can be explained by sub-diffusion rather than the boundary effect (i.e. the size of the container).

The mechanism behind the observed decrease in chromosomal mobility during embryogenesis remains an open question. This decrease did not correlate linearly with the decrease in nuclear size (Fig. 3d), indicating that this simple explanation is not feasible. However, it is possible that nuclear size affects chromosomal mobility in a non-linear manner. The formation of the nucleolus at the 8-cell stage of the *C*. *elegans* embryo has been shown to be induced by a phase separation dependent on nuclear size^19^. In this case, the increase in the density of nucleolar proteins to a threshold level induces the formation of the nucleolus. It would be intriguing to investigate whether the change in chromosomal mobility observed in the present study could also be explained by a phase separation dependent on nuclear size, as both the change in chromosomal mobility and formation of the nucleolus occur at the 8-cell stage.

## Methods

### Molecular biology and transgenic strains

Strains used in this study are listed in Supplementary Table S1 and were maintained at 22 °C or 26 °C. To visualise chromosomal loci in living cells, plasmids pKA11 [*pie-1 5*′*::gfp::lacI::pie-1 3*′ + *unc-119* ^*+*^]^36^ and pMK19A containing *lacO* repeats^10^ were co-integrated into the genome of *C*. *elegans* strain *unc-119* (*ed3*) by means of bombardment^37^. A single line showing two GFP spots in every nucleus during early embryogenesis (CAL0872) was screened.

We modified the germline fluorescence expression vectors TH312 for N-terminal mCherry fusion, TH313 for C-terminal mCherry-fusion, TH303 for N-terminal GFP fusion, and TH304 for C-terminal GFP fusion^38^ into Gateway compatible ones by means of the Gateway Vector Conversion System (Life Technologies, Carlsbad, CA, USA). In brief, an appropriate reading frame for a Gateway cassette, which is required for the Gateway recombination reaction, flanked by *att*R1 and *att*R2 sequences was blunt-end cloned into the *Sma*I sites of the multicloning sites of the original vectors. The obtained vectors were named mCherry_N_GW, mCherry_C_GW, GFP_N_GW, and GFP_C_GW, respectively.

To express mCherry::FIB-1 and mCherry::HPL-2, the open reading frames (ORFs) of *fib-1* and *hpl-2* were amplified from a *C*. *elegans* cDNA pool. The cDNAs were synthesised using a PrimeScript II 1st Strand cDNA Synthesis Kit (Takara Bio, Kusatsu, Japan) from total RNA, which was purified from N2 worms of homogeneous stages through TRIzol treatment (Life Technologies). To express mintbody for histone H4K20 mono-methylation, the 15F11scFv coding sequence was optimised for *C*. *elegans* codon usage as previously described^23^. PCR-amplified ORFs and optimised sequences, which were flanked by *att*B1 and *att*B2 sequences, were cloned into pDONR221 and replaced mCherry_N_GW and GFP_C_GW, respectively, by means of Gateway recombination cloning technology (Life Technologies). Obtained vectors were integrated into the genome of *unc-119* (*ed3*) by bombardment. A strain expressing GFP::histone H2B (CAL0231) was constructed by backcrossing the TH32 strain (GFP::histone H2B; GFP::γ-tubulin) with N2 and selecting worms expressing GFP::histone H2B but not GFP::γ-tubulin.

### Genome sequencing and assembly

Whole-genome shotgun sequencing was performed using PacBio sequencing technology. Genomic DNA was prepared from 20 plates of worm culture (9-cm dishes). Worms washed with M9 buffer were frozen at −80 °C and were ground in a bowl to a fine powder in liquid N_2_. A primary extract for genome preparation was collected by adding the buffer G2 containing RNase from the QIAGEN Genome-tip 100/G kit (QIAGEN, Venlo, The Netherlands). Purification of the genomic DNA was performed according to the manufacturer’s instructions. A 20-kb library (BluePippin size selection at 17 kb) was constructed and run on two SMRT cells in a PacBio Sequel system with a sequencing kit v1.2.1 and a DNA binding kit v1.0 (Pacific Biosciences, Menlo Park, CA, USA). The sequencing reaction generated 864,265 raw subreads with a subread N50 of 17,808 bp. *De novo* assembly was performed using the FALCON assembler (v0.3.0)^39^, and the draft assembly was polished using a resequencing algorithm (Supplementary Table S2).

### Plasmid integration site detection

To detect the integration site in the *C*. *elegans* strain CAL0872 genome, two types of plasmids (pKA11 and pMK19A) were aligned against the assembled contig sequences using the Basic Local Alignment Search Tool (BLAST)^40^. The boundary regions were amplified from genomic DNA, and products were analysed using an ABI 3730xl DNA Analyzer (Applied Biosystems, Foster City, CA, USA). The genomic position of the integration site was identified by comparison of the assembled contig and the genome sequence of *C*. *elegans* strain N2 (WBcel235).

### Live imaging

Adult hermaphrodites were dissected in M9 buffer on an 8-well slide to release embryos. Each embryo was transferred onto a 2% agarose pad mounted on a glass slide and covered with an 18 × 18 mm^2^ coverslip. The sealed slide was set on the microscopic stage. Observation of GFP or mCherry fusion proteins was performed using a CSU-X1 spinning-disk confocal system (Yokogawa, Tokyo, Japan) mounted on a BX71 microscope (Olympus, Tokyo, Japan) equipped with an UPlanSApo 100 ×/1.40 NA objective (Olympus) at 25 °C. Digital images were obtained with an iXon charge-coupled device camera (Andor Technology, Belfast, Northern Ireland) controlled by MetaMorph imaging software (Molecular Devices, Sunnyvale, CA, USA).

To analyse the mobility of the *lacO* spots, a z-series of nine planes at 2-μm intervals for 2- to 8-cell embryos and 1-μm intervals for 24- and 48-cell embryos was captured and excited at 488 nm with 150 ms of exposure for each z-plane. Three-dimensional images of nuclei were recaptured by Imaris software (Bitplane, Zurich, Switzerland), and time-dependent replacement of the *lacO* spots was tracked by means of ImarisTrack. Interphase nuclei were distinguished by determining the time course between the last and next cell divisions and the disappearance of GFP spots during mitosis.

To visualise mCherry::FIB-1 and mCherry::HPL-2, a z-series of nine planes at 2-μm intervals was captured and excited at 561 nm with 300 ms of exposure for each z-plane. Since the signals of these two proteins were dispersed during the mitotic phase^20, 41, 42^, interphase nuclei were distinguished by determining the brightness of intranuclear mCherry signals in addition to the morphologies and positions of cells and nuclei. For GFP::histone H2B (HIS-11) and 15F11scFv::GFP imaging, a z-series of 12 planes at 2-μm intervals was captured and excited at 488 nm with 100 or 200 ms of exposure. Time-lapse recordings of 1-min intervals were also performed to find nuclei in interphase based on the time course of the last and next cell divisions. Each stacked layer shown in Fig. 4, which is composed of 3–6 serial z-planes, was represented by MetaMorph imaging software.

### Data analyses (calculation of MSCD and fitting)

Based on the tracking of the two *lacO* spots in each nucleus, the distance between the two spots [*d*(*s*, *t*)] at time *t* in sample *s* was calculated. MSCD for a given time interval, *τ*, was calculated by averaging [*d*(*s*, *t* + *τ*) − *d*(*s*, *t*)]^2^ for all possible values of *s* and *t*. It should be noted that the larger *τ* is, the fewer pairs of [*d*(*s*, *t* + *τ*), *d*(*s*, *t*)] exist (Supplementary Table S3). When we fitted the MSCD vs. *τ* plot to MSCD(*τ*) = *D*_*α*_ × *τ* ^*α*^, we weighted each point by the number of pairs.

### Calculation of the theoretical distribution of the distance between two spots randomly positioned in a sphere

Using MATLAB software (MathWorks, Nattick, MA, USA), we generated a set of three random numbers uniformly distributed from −[nuclear radius] to + [nuclear radius] to create an *in silico* spot with random x-, y-, and z-coordinates within a three-dimensional sphere with a radius of that of the nucleus. If the resulting spot was located outside the sphere, the generation of a spot with random coordinates was repeated. After generating two spots with random coordinates in the sphere, the distance between the spots was calculated and recorded. This process was repeated 100,000 times to obtain the expected distribution of the distance between spots (Fig. 2, solid lines). To determine the upper and lower limits of the 95% confidence interval of the expected distance (thick and thin dotted lines in Fig. 2), the above process was repeated *N* times, in which *N* was defined as the number of pairs of *lacO* spots in Table S3 for the thick dotted lines and as the number of nuclei in Table S3 for the thin dotted lines. After repeating the simulation *N* times, a histogram of the distribution was drawn. By further repeating this *N*-times-simulation for 500 rounds, frequency values of the 13th rank (2.5%) from the top and the bottom in each bin of the histogram were identified. By connecting the top 2.5% of points and the bottom 2.5% of points, we determined the 95% confidence interval of each expected histogram in Fig. 2.

## Electronic supplementary material

Supplementary Information

Movie S1

Movie S2

Movie S3

Movie S4
