# Supplementary material for: Reduction in chromosome mobility accompanies nuclear organization during early embryogenesis in Caenorhabditis elegans
Source: Sci Rep. 2017 Jun 16;7:3631. doi: 10.1038/s41598-017-03483-5 (PMC5473868; doi:10.1038/s41598-017-03483-5)
Supplement: Supplementary file 1 — Supplementary Information [file 41598_2017_3483_MOESM1_ESM.pdf]

**Supplementary Information**

# Reduction in chromosome mobility accompanies nuclear organization during early embryogenesis in *Caenorhabditis elegans*

Ritsuko Arai, Takeshi Sugawara, Yuko Sato, Yohei Minakuchi, Atsushi Toyoda, Kentaro Nabeshima, Hiroshi Kimura, and Akatsuki Kimura

**Table S1.** ***Caenorhabditis elegans*** **strains used in this study**

**Table S2. Genome sequencing of** ***Caenorhabditis elegans*** **strain CAL0872**

**Table S3.** ***lacO*** **spot measurement summary**

**Figure S1. The genomic structure of the** ***lacO*****-inserted region in CAL0872**

PacBio sequencing revealed that the *lacO* repeat was inserted on chromosome *III*. The lower diagram plots the similarity between the sequence of chromosome *III* in CAL0872 (around 3.1 Mb) and those of the WBcel235 reference genome, pKA11 plasmid (containing *lacI*::GFP), and pMK19A plasmid (containing the *lacO* repeat). The red rectangular regions indicate the positions of the *lacO* repeat, reflecting the identical sequences of the *lacO* repeat in both the CAL0872 genome and pMK19A plasmid. In CAL0872, a 5.4-kb region (3,100,175–3,105,601 bp) of *C. elegans* chromosome *III* was replaced with a 123-kb sequence containing fragments from pKA11 and pMK19A, as well as palindromic sequences derived from the *C. elegans* genome. The deleted 5.4-kb region contains the ORFs *H06I04.1* and *Y53G8B*, which are non-essential.

**Figure S2. Estimation of the effect of phototoxicity: a comparison of chromosome mobility in the first and second halves of microscopic observation**

To exclude the possibility that the microscopic observation procedure affected the mobility of the chromosomes, changes in the distance between the two spots in the shortest observation interval (i.e. 20 s) were compared between the first and second halves of the observation. Solid lines are from the first half and dashed lines are from the second half. The distributions of the distances were roughly Gaussian, as expected, and the variances were similar between

the two halves. Therefore, the mobility of the chromosomes was unlikely to be affected by the light illumination during microscopic observation.

**Figure S3. Distribution of distances between *lacO* loci normalised by nuclear size**
(**a**) Distributions of the distances between the two *lacO* spots for each developmental stage quantified as in Fig. 2 except that the distances were normalised by the radius of the nucleus. Different colours indicate different stages. (**b**) Cumulative distribution of (**a**).

**Figure S4. Chromosomal mobility for various cell types**
The MSCDs of all cells in each stage are shown (black lines). Coloured lines and dots show MSCDs of individual cell types during the 2-cell stage (orange, P1 cells; grey, AB cells), the 4-cell stage (orange, P2 cells; blue, EMS cells; grey, ABa and ABp cells), or the 8-cell stage (orange, P2 cells; purple, C cells; blue, E cells; green, MS cells; grey, ABal, ABar, ABpl, and ABpr cells).

**Figure S5. Double logarithmic plot of MSCD**
Double logarithmic plot version of Fig. 3a to visualise that the exponent, $\alpha$, is similar among the different stages.

**Movie S1. Visualization and tracking of *lacO* spots at the 2-cell stage**
A representative movie for the tracking of the *lacO* spots at the 2-cell stage corresponding to Fig. 1b. Bar, 5 μm. Elapsed time is shown in min:sec.

**Movie S2. Visualization and tracking of *lacO* spots at the 4-cell stage**
A representative movie for the tracking of the *lacO* spots at the 4-cell stage corresponding to Fig. 1b. Bar, 5 μm. Elapsed time is shown in min:sec.

**Movie S3. Visualization and tracking of *lacO* spots at the 8-cell stage**
A representative movie for the tracking of the *lacO* spots at the 8-cell stage corresponding to Fig. 1b. Bar, 5 μm. Elapsed time is shown in min:sec.

**Movie S4. Visualization and tracking of *lacO* spots at the 48-cell stage**
A representative movie for the tracking of the *lacO* spots at the 48-cell stage corresponding to Fig. 1b. Bar, 5 μm. Elapsed time is shown in min:sec.

**Table S1. *Caenorhabditis elegans* strains used in this study**

| Strain | Genotype | Reference |
|---|---|---|
| AV221 | *unc-119(ed3) meT8 (III); meIs4* [*lac-O* + *rol-6(su1006)* + *lacO*] *meT8 (IV); meIs1*[*pie-1p::GFP::lacI* + *unc-119(+)*] | Bilgir *et al.* (2013) |
| CAL0872 | *unc-119 (ed3); wjIs95*[*pie-1p::gfp::lacI-NLS* + *unc-119(+)*]; *wjIs96*[*lacO* repeat] | This study |
| CAL0901 | *unc-119 (ed3); wjIs103*[*pie-1p::mcherry::fib-1* + *unc-119(+)*] | This study |
| CAL0911 | *unc-119 (ed3)*; *wjIs104*[*pie-1p::mcherry::hpl-2* + *unc-119(+)*] | This study |
| CAL0921 | *unc-119 (ed3); wjIs105*[*pie-1p::15F11scFV::gfp* + *unc-119(+)*] | This study |
| CAL0231 | *unc-119 (ed3); ruIs32*[*pie-1p::gfp::histone H2B(his-11)* + *unc-119(+)*]*III* | This study |

**Table S2. Genome sequencing of *Caenorhabditis elegans* strain CAL0872**

| Data set / Assembly | | Total bases (bp) | Total number | Minimum length (bp) | Maximum length (bp) | N50 (bp) | Mean (bp) |
|---|---|---|---|---|---|---|---|
| PacBio subreads | | 9,816,451,169 | 864,265 | 50 | 74,577 | 17,808 | 11,358 |
| FALCON (≥15 kb) | Primary | 101,577,359 | 91 | 18,382 | 5,018,309 | 2,274,662 | 1,116,014 |
| | Alternative | 439,339 | 15 | 19,293 | 50,678 | 27,673 | 29,289 |

**Table S3. The *lacO* spot measurement summary**

| | 2cell | 4cell | 8cell | 24cell | 48cell |
|---|---|---|---|---|---|
| **radius [µm]** | 4.08 | 3.60 | 3.16 | 2.52 | 2.12 |
| **the number of nuclei** | 37 | 44 | 42 | 19 | 48 |
| **the number of pairs of the *lacO* spots** | 889 | 1,017 | 1,113 | 475 | 1,237 |
| **the total number of pairs of distance changes** | 10,800 | 11,753 | 14,656 | 5,700 | 15,429 |
| **the number of pairs of distance changes** | | | | | |
| **(interval = 20 s)** | 852 | 973 | 1,071 | 456 | 1,189 |
| **(interval =40 s)** | 815 | 929 | 1,029 | 437 | 1,141 |
| **(interval =60 s)** | 778 | 885 | 987 | 418 | 1,093 |
| **(interval =80 s)** | 741 | 841 | 945 | 399 | 1,045 |
| **(interval =100 s)** | 704 | 797 | 903 | 380 | 997 |
| **(interval =120 s)** | 667 | 753 | 861 | 361 | 949 |
| **(interval =140 s)** | 630 | 709 | 819 | 342 | 901 |
| **(interval =160 s)** | 593 | 665 | 777 | 323 | 853 |
| **(interval =180 s)** | 556 | 621 | 735 | 304 | 805 |
| **(interval =200 s)** | 519 | 577 | 693 | 285 | 757 |
| **(interval =220 s)** | 482 | 533 | 651 | 266 | 709 |
| **(interval =240 s)** | 445 | 489 | 609 | 247 | 661 |
| **(interval =260 s)** | 408 | 445 | 567 | 228 | 613 |
| **(interval =280 s)** | 371 | 401 | 525 | 209 | 565 |
| **(interval =300 s)** | 335 | 357 | 483 | 190 | 517 |
| **(interval =320 s)** | 302 | 316 | 441 | 171 | 469 |
| **(interval =340 s)** | 269 | 275 | 399 | 152 | 421 |
| **(interval =360 s)** | 236 | 239 | 357 | 133 | 373 |
| **(interval =380 s)** | 203 | 206 | 315 | 114 | 325 |
| **(interval =400 s)** | 172 | 174 | 273 | 95 | 277 |
| **(interval =420 s)** | 148 | 143 | 232 | 76 | 230 |
| **(interval =440 s)** | 125 | 115 | 193 | 57 | 183 |
| **(interval =460 s)** | 103 | 91 | 160 | 38 | 137 |
| **(interval =480 s)** | 82 | 71 | 132 | 19 | 93 |
| **(interval =500 s)** | 63 | 53 | 107 | | 55 |
| **(interval =520 s)** | 52 | 40 | 86 | | 33 |
| **(interval =540 s)** | 42 | 27 | 72 | | 21 |
| **(interval =560 s)** | 34 | 16 | 60 | | 12 |
| **(interval =580 s)** | 26 | 9 | 49 | | 5 |

| | | | |
|---|---|---|---|
| **(interval =600 s)** | 18 | 3 | 39 |
| **(interval =620 s)** | 12 | | 30 |
| **(interval =640 s)** | 8 | | 23 |
| **(interval =660 s)** | 5 | | 16 |
| **(interval =680 s)** | 3 | | 10 |
| **(interval =700 s)** | 1 | | 4 |
| **(interval =720 s)** | | | 2 |
| **(interval =740 s)** | | | 1 |

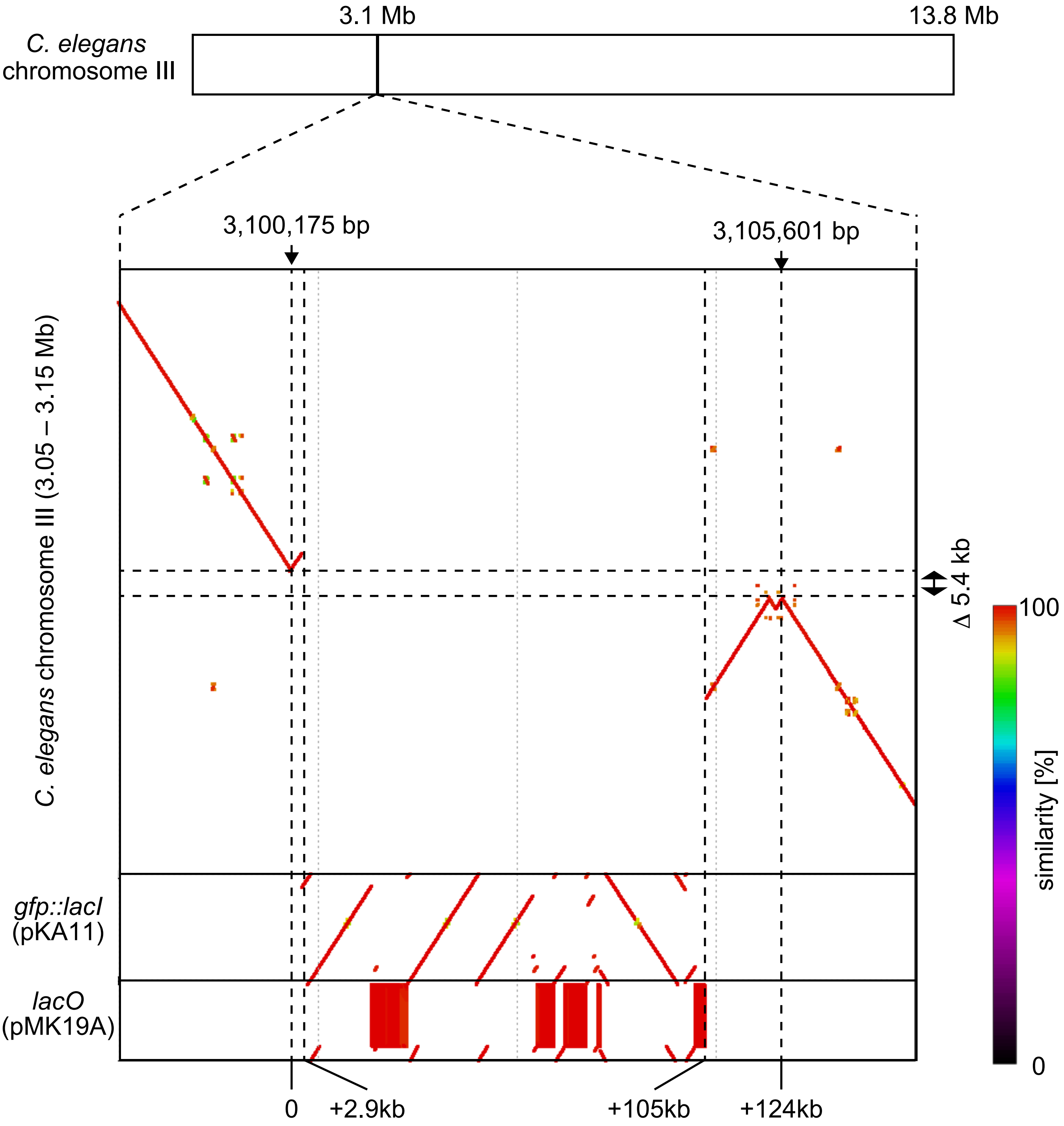


# Arai et al. Figure S1

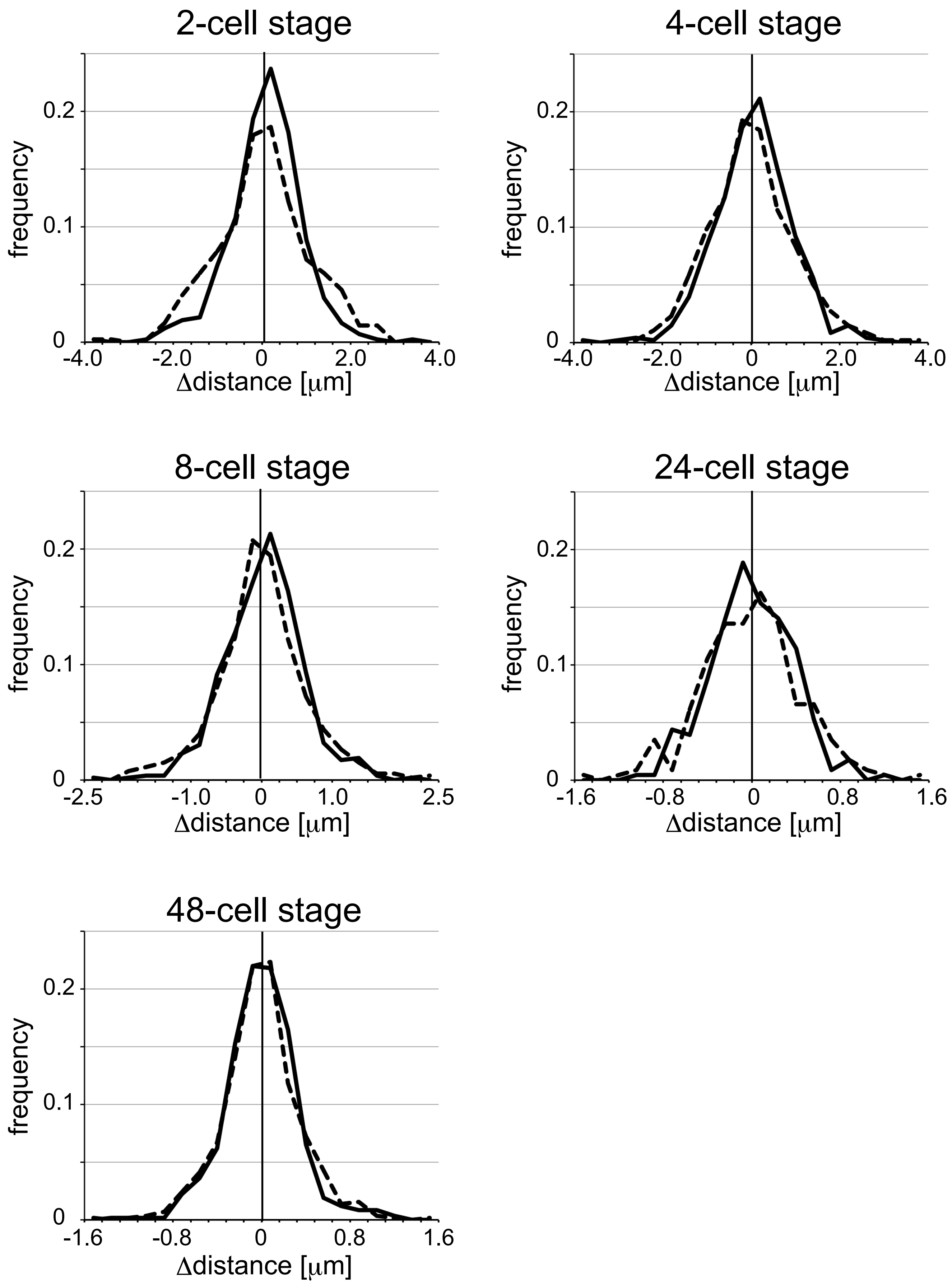


Arai et al. Figure S2

**a**

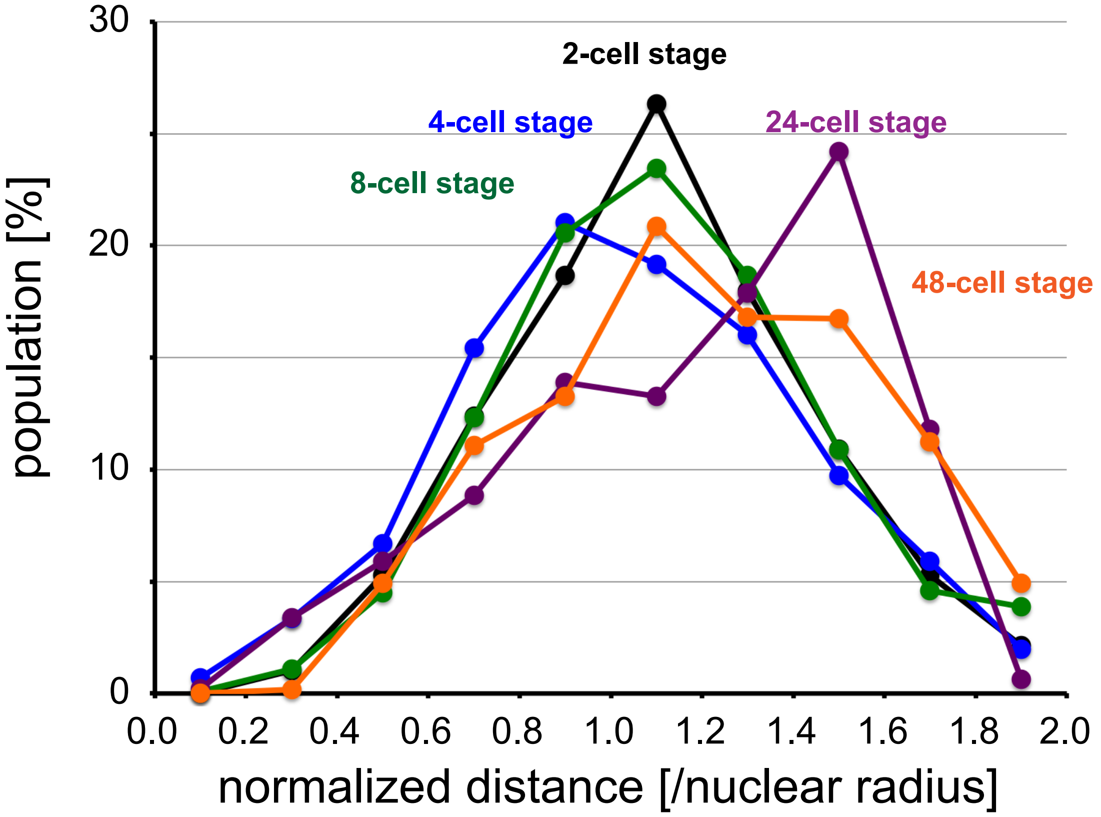


**b**

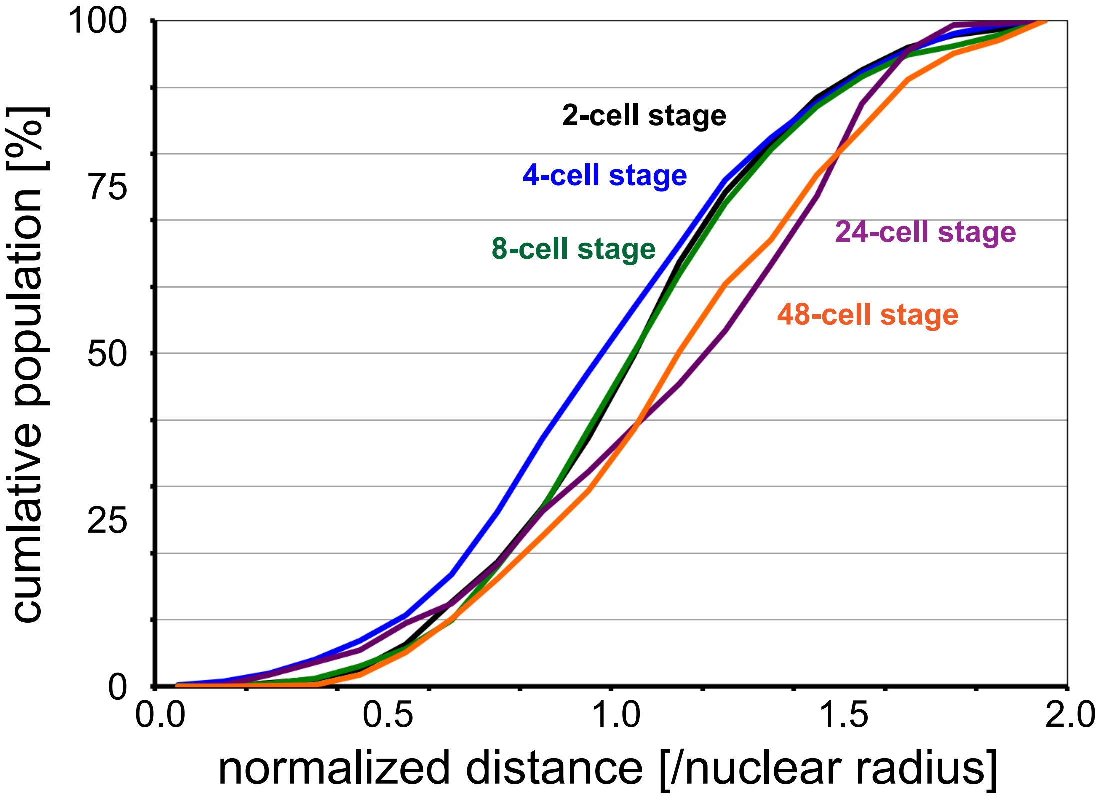


Arai et al. Figure S3

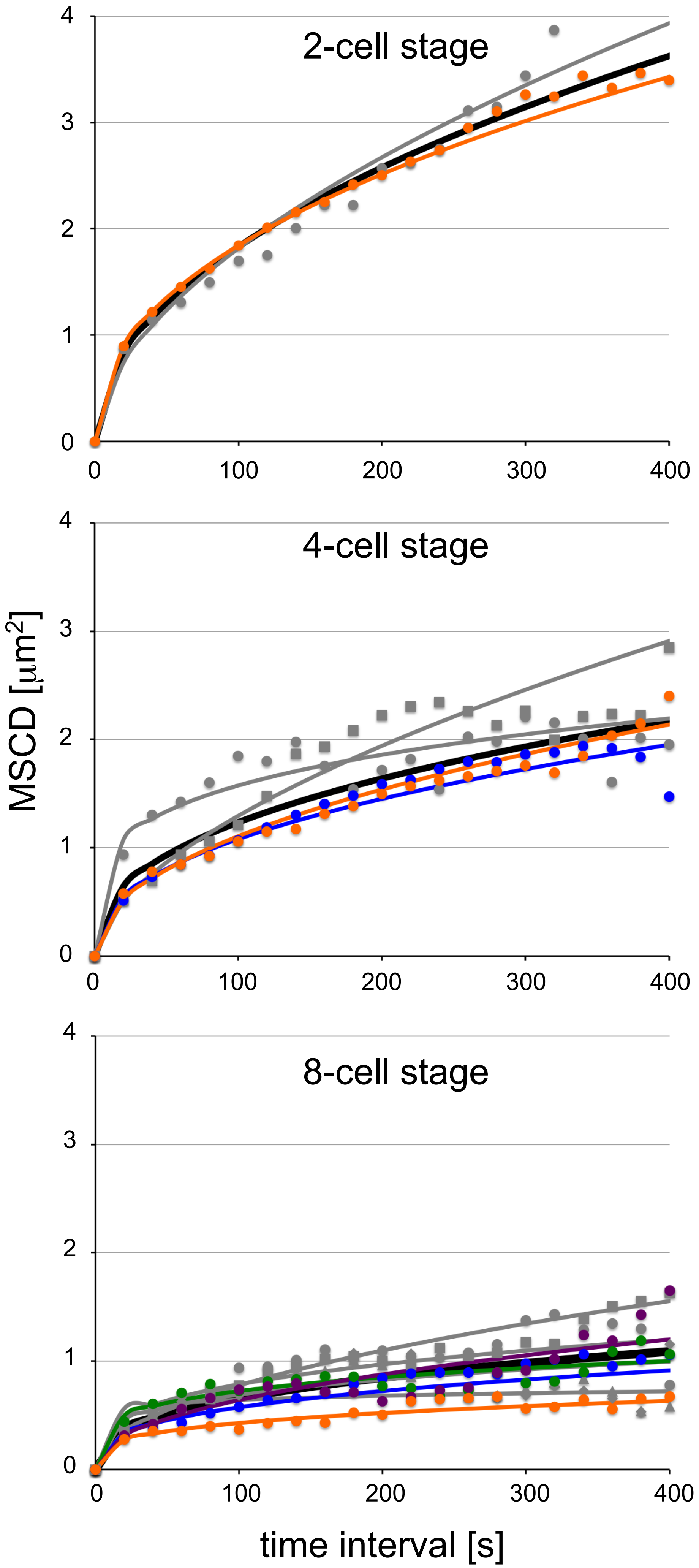


Arai et al. Figure S4

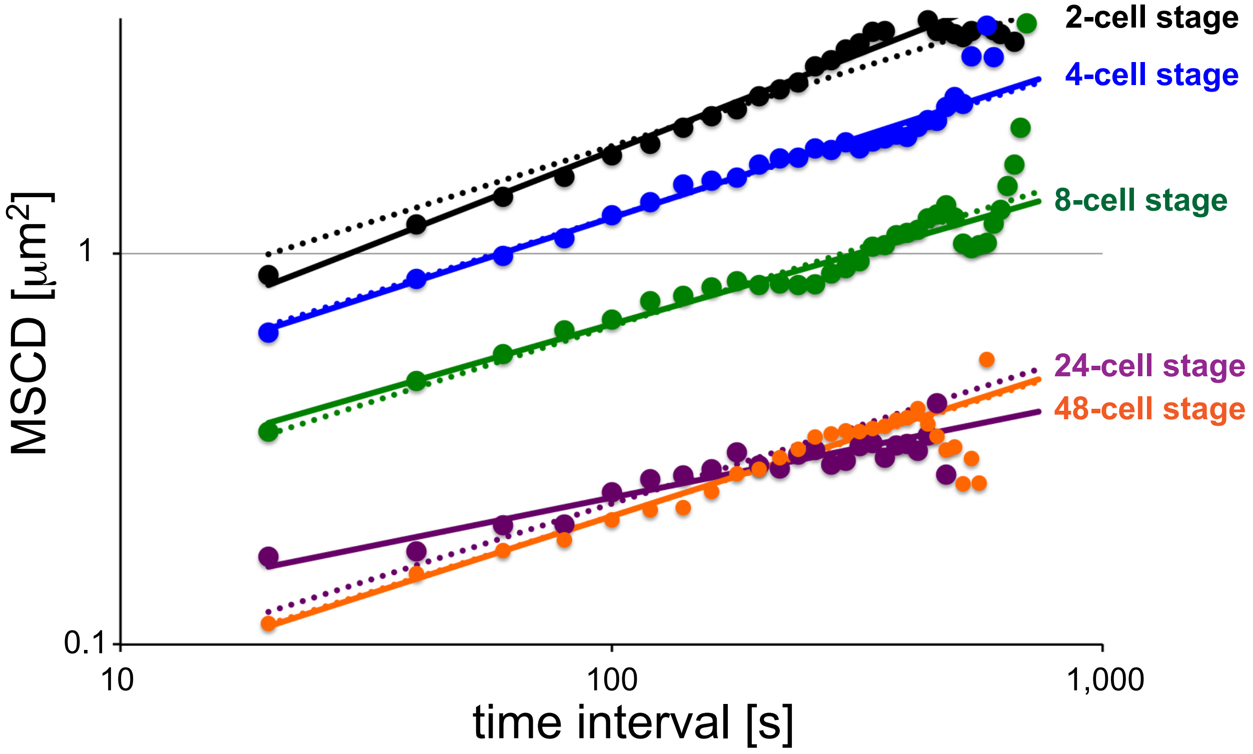


Arai et al. Figure S5
